# Knowledge of Obstetric Danger Signs among Pregnant Women in the Eastern Democratic Republic of the Congo

**DOI:** 10.3390/ijerph20085593

**Published:** 2023-04-20

**Authors:** Bin-Eradi Imani Ramazani, Simon-Decap Mabakutuvangilanga Ntala, Daniel Katuashi Ishoso, Monique Rothan-Tondeur

**Affiliations:** 1 Nursing Sciences Research Chair, Laboratory Educations and Health Practices (LEPS), (EA 3412), UFR SMBH, University Paris, Sorbonne Paris Cite, F-93017 Bobigny, France; decapntela@gmail.com (S.-D.M.N.); rothan-tondeur@univ-paris13.fr (M.R.-T.); 2 Nursing Sciences Section, Institut Supérieur des Techniques Médicales de Kindu (ISTM-KINDU), PB.304, Kindu P.O. Box 9912, Democratic Republic of the Congo; 3 Center for Research in Nursing Sciences and Health Innovation (CReSIIS), K-012, Kinshasa P.O. Box 11850, Democratic Republic of the Congo; dishosok@gmail.com; 4 Section of Nursing Sciences, Institut Supérieur des Techniques Médicales de Kinshasa, BP 774 Lemba, Kinshasa P.O. Box 11850, Democratic Republic of the Congo; 5 Department of Community Health, Kinshasa School of Public Health, University of Kinshasa, Kinshasa P.O. Box 11850, Democratic Republic of the Congo; 6 Assistance Publique-Hôpitaux de Paris (AP-HP), Nursing Sciences Research Chair, F-75005 Paris, France

**Keywords:** knowledge evaluation, pregnant women, obstetric danger signs, Democratic Republic of the Congo

## Abstract

A lack of awareness regarding obstetric danger signs (ODS) is one of the factors that delay a pregnant woman’s decision to seek emergency obstetric care. In developing countries, this delay can lead to high morbidity and mortality among pregnant women. In eastern Democratic Republic of Congo (DRC), very few studies have been conducted to assess the level of knowledge of pregnant women about ODS. Therefore, this study aimed to assess the knowledge of pregnant women about ODS in health facilities in eastern DRC. This quantitative cross-sectional, descriptive, and analytical study was conducted in 19 health facilities in the Kasongo health zone in the south Maniema Province of eastern DRC. A total of 624 pregnant women aged 12–49 years were interviewed in this study. Of these, 60.6% were secondary school graduates, >99% were married, 85.5% were cultivators, and 67.9% were Muslims. The knowledge of ODS among pregnant women was low (21.9%). The most cited danger signs during pregnancy, labor/delivery, and postpartum included severe abdominal pain and severe vaginal bleeding. Additionally, pregnant women aged 30–39 years (*p* = 0.015) and those who had given birth once (*p* = 0.049), twice (*p* = 0.003), 3–5 times (*p* = 0.004), and >5 times (*p* = 0.009) were more likely to be aware of ODS than others. Our findings indicated that pregnant women have little knowledge of ODS, which makes it difficult for them to take prompt decisions to seek emergency obstetric care. Thus, strategies to increase the knowledge of pregnant women about obstetrical danger signs by healthcare providers during prenatal consultations (antenatal care) must be developed to improve their rapid decision-making skills during pregnancy, labor, and postpartum.

## 1. Introduction

In developing countries, childbirth is often associated with life-threatening obstetric complications during pregnancy, labor/delivery, and postpartum [1]. These obstetrical complications are sometimes unpredictable and can occur at any time during pregnancy [2].

In 2017, 295,000 women died during pregnancy, labor/delivery, and postpartum, and 94% of these women belonged to developing countries [3]. While all member states, under the auspices of the United Nations, have set a goal of reducing the maternal mortality ratio to less than 70 per 100,000 live births in 2030 [4]. Studies have revealed that a majority of women, men, and community members have little knowledge of ODS [1,2,5,6,7,8]. In the Democratic Republic of the Congo (DRC) and other developing countries, this lack of knowledge among all citizens is one of the factors that can delay the decision of the pregnant woman or her family to seek emergency obstetric care [2,3,5,7,8].

ODS are unexpected situations that occur in pregnant women during pregnancy, labor/delivery, or postpartum. Such situations account for >75% of maternal deaths in developing countries [9,10,11,12,13]. The knowledge and understanding of pregnant women about ODS are critical in making real-time decisions to seek emergency obstetric care [7]. Effective strategies to prevent childbirth complications include health promotion education for women, men, and community members to increase awareness about ODS [14].

To this end, the World Health Organization (WHO) recommends providing information on ODS to all pregnant women at each prenatal visit [15]; this will enable the early identification of ODS and facilitate appropriate emergency obstetric care of pregnant women [16].

In the DRC, as per the maternal death notification system, >32% of maternal deaths in 2018 were related to delays in seeking obstetric care [17]. More recently, a 2021 study in the Maniema Province of eastern DRC estimated that the maternal mortality ratio was 620 deaths per 100,000 live births, with 46% of these deaths attributed to the first delay in decision-making by the pregnant woman or her family to visit the place of delivery during the first uterine contractions [18]. According to Nkamba et al. [2], pregnant women’s lack of awareness about and poor understanding of the ODS and erroneous advice given by some healthcare providers during antenatal consultation (ANC) sessions can delay seeking emergency obstetric care, thereby increasing the risk of maternal deaths [2].

However, maternal mortality rates can be reduced if pregnant women are made aware of the ODS, which will prompt them to seek emergency obstetric care. Thus, this study aimed to assess the knowledge of ODS among pregnant women in health facilities in eastern DRC.

## 2. Materials and Methods

### 2.1. Study Design

This is a quantitative, cross-sectional, analytical study assessing pregnant women’s knowledge of ODS, conducted from September to December 2021.

### 2.2. Setting

This study was conducted in the southern Maniema Province of eastern DRC in the Kasongo health zone (HZ). A total of 19 health facilities from the state sector were included in this study, including 17 health centers and 2 reference health centers. Maniema is 1 of the 26 provinces in DRC and is located in eastern DRC. It has an estimated population of 2,938,101 citizens, with women of childbearing age (15–49 years) accounting for 21% (n = 617,001) of the total population, whereas 4% (n = 117,524) of the total population is expected to comprise pregnant women [19].

A DRC health system HZ is a decentralized entity that is responsible for the planning and development of health activities for primary healthcare. It is supervised at the intermediate level [20] and operates according to the strategies, norms, and directives decreed by the central health legislative body [21]. A single HZ can have a population of approximately 50,000–100,000 inhabitants in rural areas and approximately 100,000–250,000 in urban areas [19].

Our study was conducted at the Kasongo HZ located in the southern Maniema Province. The estimated population of this area is 265,693 individuals, with 55,796 (21%) women of childbearing age. The expected number of pregnant women per year is 4% (10,628) of the general population [19].

We selected Kasongo HZ as the study site based on the results of a previous study on maternal mortality conducted in eastern DRC in three HZs—Kasongo, Kunda, and Kibombo—in southern Maniema Province. The previous study revealed that the Kasongo HZ had a higher maternal mortality rate, estimated at 698 deaths per 100,000 live births, than the other 2 HZs, which had 612 deaths per 100,000 live births in the Kunda HZ and 513 deaths per 100,000 live births in the Kibombo HZ [18].

### 2.3. Study Population and Sampling

The study population comprised pregnant and lactating women with children aged < 3 months who were registered in the prenatal consultation registers of the health facilities in the Kasongo HZ were present during the study period and agreed to participate in the survey.

### 2.4. Sampling

#### 2.4.1. Sample Size

The minimum sample size was calculated to be 288 as follows:

(1) $$n\geq \frac{Z^{2}\alpha .p.q}{d2}$$

where p represents the proportion of pregnant women with an acceptable level of knowledge about ODS of 25% [1]; q denotes complement of p = 1 − p, q = 50%; z denotes the 95% confidence level (CI) value according to the normal distribution of data (1.96); and d denotes the degree of precision (5%).

Considering a nonresponse rate of 10%, the sample size was increased to 317. However, this number was multiplied by two to increase precision by reducing the margin of error. Following the logic used by Bintabara [1], the final sample size was estimated at 634.

#### 2.4.2. Sampling Technique

Considering the health areas as population clusters, we conducted stratified sampling in proportion to the population size as follows: the population was divided into subpopulations as per clusters that comprise health areas; the proportion of each health area (cluster) was calculated based on the total population, i.e., the number of pregnant women expected per area divided by the total number of subjects in the HZ. The resulting proportions were multiplied by the sample size to obtain the subsamples of each health facility.

Given pregnant women are registered with prenatal consultation registers, a systematic random sampling method was used to select the participants to be interviewed from each health facility. Thus, sampling was performed by dividing the total number of participants by the sample size of the health facility. The first participant was randomly selected between Participant #1 in the ANC register and the participant corresponding to the sampling step; the other participants were subsequently selected each time by repeating the sampling step.

### 2.5. Data Collection Techniques

To collect the data for this study, a questionnaire was designed with reference to the study [6], which was contextualized, pre-tested, and readjusted.

### 2.6. Study Variables

#### 2.6.1. Definition of Variables

ODS included severe vaginal bleeding, fever, prolonged fatigue, abdominal pain, fetal disappearance or decreased movement, difficulty in breathing, severe headache or blurred vision, convulsions/loss of consciousness, foul-smelling vaginal discharge, membrane rupture before the onset of labor, prolonged labor of >12 h, swelling of the hands or face, and placental retention; knowledge of these was considered the dependent variable. Sociodemographic and clinical characteristics, including age, religion, education level, marital status, parity, gestational age, and ANC, were considered independent variables. The study variables are defined in Table 1.

#### 2.6.2. Operational Definitions of the Variables

The dependent variable response was dichotomous (yes vs. no). Thus, any woman who could name at least three were considered knowledgeable [6,14,21]. Those who cited none, one, or two signs were considered to lack knowledge about ODS. The knowledge of pregnant women increased as the stages of pregnancy progressed: ante-, peri-, and postpartum. The operational definitions of the variables are presented in Table 2.

### 2.7. Data Collection Tool

Structured interviews were conducted to collect information about pregnant women’s knowledge of ODS. Survey questionnaires were created on the CoBoCollect platform, followed by downloading, installing, and configuring the CoBoCollect mobile application on the interviewers’ tablet devices to begin data collection [22]. Five nurses with experience in reproductive health and primary care who could speak the local language (Swahili) were selected. They were trained for 2 days by the principal investigator on conducting the structured interviews and using the data collection tool (KoboCollect^®^ v2022.4.4, including installation in the tablet device, data recording, and transmission to the central data collector).

The data collection tool included three parts: sociodemographic characteristics, clinical characteristics, and notions about obstetric complications ante-, peri-, and postpartum.

Prior to the actual survey, a pilot survey was conducted on 31 participants. The survey results were not included in the final study database. This pilot survey enabled us to ensure the interviewers’ efficiency of the KoBoCollect^®^ tool, test the participants’ comprehension of the questionnaire, estimate the data collection duration, judge its adequacy in relation to the context of this study, and determine the feasibility of analyses at all stages.

### 2.8. Data Analysis

All data were analyzed using STATA version 15.0 (StataCorp LLC., College Station, TX, USA) [23]. All parameters of central tendency and dispersion were considered quantitative variables. Qualitative variables were expressed as absolute numbers and frequencies. Quantitative data with normal distribution were expressed as mean (± standard deviation), and those with nonnormal distribution were expressed as the median (interquartile range). Categorical variables were summarized as proportions and presented in tables or graphs. Bivariate analyses using the Chi-square test and multivariate analysis were conducted to determine the association between the dependent and independent variables. For multivariate analysis, forward stepwise logistic regression was used to identify independent predictors. Only variables with significant association in the bivariate analysis were included in the final model. The strength of association was quantified in terms of the odds ratio (OR) with a corresponding 95% confidence interval. A *p*-value of <0.05 was considered statistically significant.

### 2.9. Ethical Considerations

The protocol for this study had previously been submitted to the National Ethics Committee of the DRC, which had issued a favorable opinion in its decision N°0280/CNES/BN/PMMF/2021 on 26 August 2021. Approval was also sought and obtained from the Head of the Provincial Health Division of Maniema (N°DPS 53.02/B.CD-MMA/SEC/MMS/907/2021). Interviews with participants were conducted at a location of their choice. The research team obtained written informed consent before each interview. For participants who were minors (aged 15–18 years), written informed consent was obtained from the respondent as well as from the respondent’s parent or legal guardian. Before signing, the researchers read the informed consent form aloud and explained in detail all the important aspects of this study, including the objective, interest, purpose, and interview procedures. The women were also informed that participation in this study was entirely voluntary. Furthermore, anonymity and confidentiality were guaranteed to survey participants during all stages of data handling.

## 3. Results

### 3.1. Description of the Population

The study population comprised pregnant women, parturients, and women who had given birth and were registered with the ANC registers of the Kasongo HZ health structures.

### 3.2. Sociodemographic and Clinical Characteristics of the Respondents

A total of 634 pregnant women were approached in the Kasongo HZ out of the expected 10,628 in 2021, with 626 women responding favorably, thus yielding a response rate of 98.4% and corresponding to 6% of the population concerned. The average age was 25 years, and the majority of women were aged 20–29 years. More than half of the respondents (60.6%) had attended high school, >99% were married or in a common-law relationship, a large majority were farmers or traders, and the majority (67.9%) were Muslim. In addition, most respondents had given birth at least once in the past and had visited a health center for ANC. However, only 10.1% of them had attended all 4 recommended ANC sessions. Table 3 presents the sociodemographic and clinical characteristics of the respondents.

In terms of knowledge of ODS among women in the Kasongo HZ, 21.9% (137/626) of the women had good knowledge about ODS. Table 4 summarizes the knowledge of women with respect to ODS.

### 3.3. Frequency of ODS Cited during Pregnancy (Antepartum)

**Figure 1:**
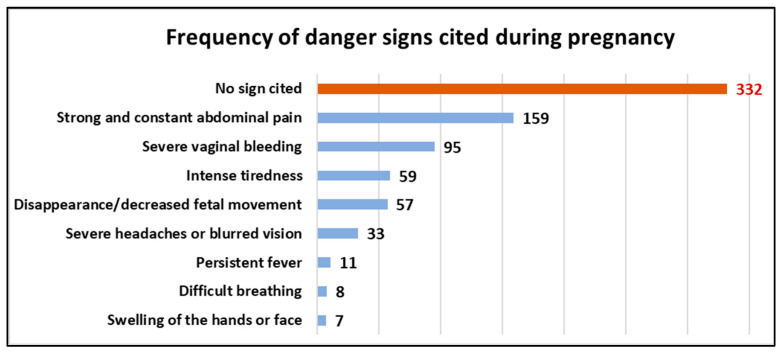
Frequency of ODS cited during antepartum.

Of the 626 women surveyed, 332 (53%) did not cite ODS during pregnancy. However, constant severe abdominal pain was the most frequently cited obstetric danger sign by 159 (25.3%) participants. Severe headache or blurred vision, persistent fever, difficulty in breathing, and swelling of the hands or face were the least cited ODS, cited by 33 (5.2%), 11 (1.7%), 8 (1.2%), and 7 (1.1%) participants, respectively, during this period. Figure 1 presents the frequency of the ODS cited during the antepartum period.

### 3.4. Frequent ODS Cited during Labor or Delivery (Peripartum)

**Figure 2:**
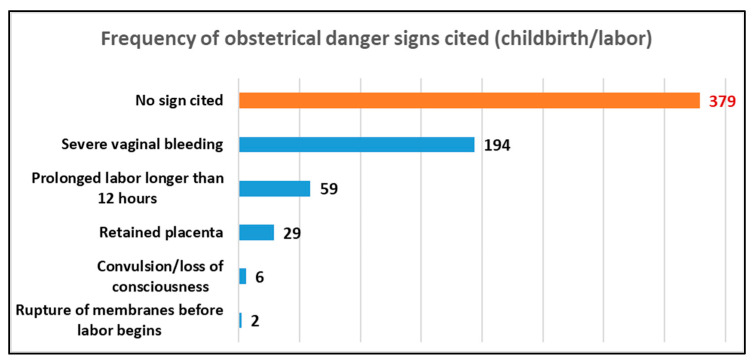
Frequency of ODS cited during peripartum (childbirth/labor).

Of the 626 participants, 379 (60.5%) did not cite any ODS during the peripartum period. However, severe vaginal bleeding was the most cited obstetric danger sign cited by 194 (31%) participants. Convulsions or loss of consciousness and rupture of the membrane before the onset of labor were cited by 6 (0.9%) and 2 (0.3%) participants, respectively, making these the least cited ODS during the peripartum period. Figure 2 presents the frequency of the ODS cited during the peripartum period.

### 3.5. Frequency of ODS Cited after Delivery (Postpartum)

**Figure 3:**
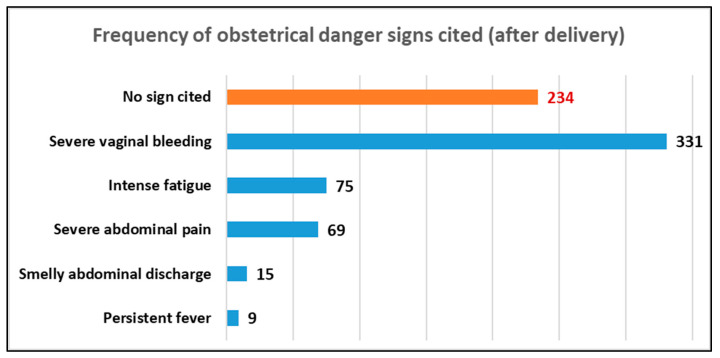
Frequency of ODS cited during postpartum (after delivery).

Overall, 331 (52.8%) respondents cited severe vaginal bleeding as an obstetric danger sign. However, more than one-third (37.3%) of these women did not cite any ODS during the postpartum period. Foul-smelling vaginal discharge and persistent fever were the least cited ODS during this period, cited by 15 (2.3%) and 9 (1.4%) participants. Figure 3 presents the frequency of the ODS cited during the postpartum period.

Regarding the factors affecting the knowledge of ODS among women, the bivariate analysis revealed that pregnant women aged 30–39 years (adjusted OR [ORa] = 2.4, CI: 1.3–4.2), and those who delivered twice (ORa = 4.5, CI: 1.7–12.5), 3 to 5 times (ORa = 4.7, CI: 1.8–12.6), and more than 5 times (ORa = 5.4, CI: 2.1–14.0) were more likely to be aware of ODS than other women.

In contrast, the final logistic regression model on knowledge of ODS as a function of independent variables revealed that pregnant women who delivered once (ORa = 2.9, CI: 1.0–8.1), twice (ORa = 5.4, CI: 1.8–16.6), 3 to 5 times (ORa = 5.3, CI: 1.7–16.9), and more than 5 times (ORa = 4.9, CI: 1.5–16.4) were likely to have better knowledge about ODS than others, after adjusting for independent variables. Table 5 presents the knowledge about ODS according to the sociodemographic and clinical characteristics of the respondents, and Table 6 presents the final logistic regression model of knowledge of ODS according to independent variables.

## 4. Discussion

This study aimed to assess the knowledge of ODS among women in the health facilities of Kasongo HZ in the southern Maniema Province of eastern DRC.

### 4.1. Knowledge of ODS among Women

The majority of preventable maternal deaths in developing countries are due to delays in the pregnant woman’s or her family’s decision to seek care, delays in visiting the place of delivery, and delays in receiving adequate hospital care [22]. In such cases, the lack of awareness about ODS is a major contributor to delays in seeking emergency obstetric care, resulting in high maternal mortality and morbidity rates [24].

Our study revealed that the overall knowledge of ODS among women was 21.9%. This is consistent with the value reported by a study conducted in Southern Ethiopia in Yirgacheffe City (21.9%) [14] but higher than that reported in Jordan (15.2%) [25] and Wolaita Sodo City in Southern Ethiopia (16.8%) [4]. In addition, it is lower than the values reported by studies conducted in Chamwino District, Tanzania (25%) [1], Angolela Tera District in Northern Ethiopia (37.5%) [6], Shashamane town in the Oromia Region in Ethiopia (40%) [8], and Kwazulu Natal in South Africa (52%) [26]. This difference is because of the differences in the implementation of reproductive health programs, particularly in the organization of ANC services in these different countries, as well as the different sociocultural contexts that characterize each of these regions [1,7].

### 4.2. ODS Most Cited by Respondents

In our study, ODS were classified into three periods: ante-, peri-, and postpartum. The most frequently cited antepartum danger sign was severe abdominal pain, which is contradictory to that reported by Nkamba et al., who found that vaginal bleeding was the best-known danger sign [2]. Other studies in the African region, such as those conducted by Bintabara in Tanzania and Hibstu and Woldeamanuel in Ethiopia, revealed that severe vaginal bleeding was the most frequently cited antepartum obstetric danger sign [1,6,14]. Further, severe vaginal bleeding was the most frequently cited obstetric danger sign during the peri- and postpartum periods. These results are consistent with that of studies by the researchers above. Severe vaginal bleeding may be perceived as a clearly abnormal sign by women [1,6,14].

We agree with previous reports stating that intense abdominal pain and severe vaginal bleeding in women during the ante-, peri-, and postpartum periods can be perceived as abnormal signs and attract their attention.

### 4.3. ODS Least Cited by Respondents

Pre-eclampsia is recognized as the second leading cause of maternal mortality [1]. However, symptoms that accompany this obstetric complication, such as severe headache/blurred vision, swelling of the hands or face, and convulsions or loss of consciousness, were the least cited danger signs cited by our respondents during the ante- and peripartum periods. These results are consistent with those reported by Nkamba in the DRC [2]; Bintabara in Chamwino District, Tanzania; Mbalinda at the Mulago Hospital in Uganda; and Kaso in Robe Woreda, Arsi Zone, Oromia Region, Ethiopia. These authors reported that there is low awareness of ODS during ANC sessions [1,6,14]. Nkamba stated that signs such as headache and extreme fatigue may be misinterpreted as normal signs of pregnancy by pregnant women without considering any obstetric complications; thus, the study suggested caregivers advise pregnant women who regard these obstetric complications as normal situations during ANC sessions [2].

Although we agree with the perceptions of these authors, we believe that the participation of women in ANC sessions was low in those studies, as our study results revealed that >43% of our respondents did not participate in the first ANC session. Furthermore, only 10.1% of them attended all 4 recommended ANC sessions. Therefore, women must ensure participation in all four ANC sessions to receive adequate information on ODS and benefit from comprehensive health education on childbirth preparation [1,27].

### 4.4. Factors Associated with the Knowledge of ODS among Pregnant Women

Our study revealed a significant association between marital age and knowledge of ODS among women. Women aged 30–39 years were more likely to be aware of ODS than those in other age groups (*p* ˂ 0.015). This result contradicts those reported by studies conducted in Yirgacheffe town, Gedeo Zone, Southern Ethiopia by Bolanko; Wolaita Sodo town, Southern Ethiopia by Hibstu; Southeastern Nigeria by Ossai; and Arba Minch town, Central Ethiopia by Workineh. These studies stated that the knowledge of ODS was likely to be higher in the age group of 25–34 years. Women in this age category are believed to be physically and psychologically ready to accept information about ODS [7,12,14,24].

However, based on our findings, we believe that most women in the age group of 30–39 years have given birth several times, which may serve as an experience in learning more about ODS.

### 4.5. Knowledge of ODS and Respondents’ Education Levels

Our study revealed that there was no significant difference between the education level of women and knowledge of ODS, although >93% of the respondents had an acceptable education level (primary, secondary, or university level). However, an educated woman may be better informed and take autonomous decisions about her health [7,8,13,14]. Our results are contradictory to those reported by Bintabara in Tanzania, Hibstu and Wassihum in Northern Ethiopia, and Bolanko in Southern Ethiopia, who stated that women with primary, secondary, and university levels of education were more likely to be well informed about ODS than those without education. They stated that a woman with a good formal education can effectively improve her knowledge and health behaviors and take prompt action when ODS begins to appear [1,7,8,14].

We believe that our results are attributable to the low level of participation in ANC sessions in our study as only a minority (10.1%) of our respondents who had given birth at least once had attended the 4 recommended ANC sessions. Pregnant women should attend more ANC sessions so that they can be counseled on the ODS during the ante-, peri-, and postpartum periods [7,28,29].

### 4.6. Knowledge of ODS and ANC Follow-Up

Although more than half (56.7%) of our respondents who had given birth at least once visited the hospital for ANC sessions, our study revealed that the measures taken to combat maternal mortality in the DRC using the ANC approach are lacking, which is the most important strategy implemented by the Ministry of Public Health through the national reproductive health program to reduce maternal mortality [30]. No significant difference was found between the number of ANC visits and knowledge of ODS. These results are consistent with the findings reported by Bintabara in Chamwino District, Tanzania, which did not identify an association between the number of ANC visits and knowledge about ODS [1]. Conversely, another study by Wassihun in Schamane, Oromia Region, Ethiopia, found a significant association between the aforementioned two parameters. Furthermore, Migliani indicated that although the vast majority of pregnant women (90%) in the DRC participated at least once in an ANC session, they did not sufficiently benefit from information on obstetric complications [30]. In addition, Mbalinda reported that pregnant women who are aware of ODS are more likely to be knowledgeable about preparations for birth and delivery and obstetric complications in the ante-, peri-, and postpartum periods than those who are completely unaware of such signs [31].

In this regard, the WHO recommends providing information on ODS to all pregnant women at every prenatal visit [15] to enable the early identification of ODS and to facilitate appropriate emergency obstetric care [16].

We believe that the themes included in the DRC’s national reproductive health program on ANC should be updated by incorporating ideas about obstetric complications that can occur among pregnant women during the peri- and postpartum periods. Further, qualitative studies are warranted to better understand this phenomenon (i.e., the mismatch between the number of ANC visits and pregnant women’s knowledge of ODS) in our study.

### 4.7. Knowledge of ODS and Parity of Respondents

We found that after adjusting for independent variables, the knowledge of ODS among women was significantly associated with parity. The knowledge increased significantly for pregnant women who delivered once twice, three to five times, and more than five times. Our results contradict those reported by previous studies conducted in other African countries, where knowledge of ODS was significantly associated with pregnant women who delivered three to five times [6,7,32]. However, we agree with these authors that this could be due to their prior experiences with obstetric complications during the ante-, peri-, and postpartum periods. They may have received information from their social community (old women and traditional birth attendants) [7,32,33].

### 4.8. Strengths and Limitations of the Study

To the best of our knowledge, this is the first study to assess pregnant women’s knowledge of ODS conducted in the DRC. Through this study, we were able to determine women’s knowledge of obstetric complications that can occur during the ante-, peri-, and postpartum periods. In addition, our findings revealed weaknesses in the measures taken to reduce maternal mortality in the DRC through the ANC approach [34]. However, a limitation of this study is that we did not consider pregnant women who have already developed complications in the past. An additional element would be to determine the association between women’s knowledge of ODS and the variable of women who have or have not experienced these signs during the ante-, peri-, or postpartum period. However, this limitation was mitigated by including women who had previously given birth and were breastfeeding during the survey.

## 5. Conclusions

The knowledge of ODS among women remains low (21.9%). We found a mismatch between the number of ANC visits and the knowledge of ODS among pregnant women. This is a significant barrier to reducing maternal mortality by making prompt decisions to seek emergency obstetric care. However, proper education of pregnant women improves their knowledge of obstetric danger signs and could promote timely consultation and the use of modern health services. Therefore, we believe that the themes included in the strategies implemented by the DRC’s national reproductive health program on ANC should be updated to incorporate ideas about obstetric complications that can occur during the ante-, peri-, and postpartum periods of pregnancy. Furthermore, caregivers must develop strategies to improve pregnant women’s knowledge of ODS during ANC sessions in order to improve their rapid decision-making in the three periods. Evaluating the knowledge of the caregivers who sensitize these women during ANC would also be necessary to determine their true knowledge level on the ODS so that the DRC’s national reproductive health program could properly customize ANC content. Finally, qualitative research is needed to understand pregnant women’s delayed decision to use modern health services in early labor.

## Figures and Tables

**Table 1:** Presentation of variables.

Variables	Definitions	Types	Modalities	Justifications
Sociodemographic characteristics				
Age	Age range of pregnant women aged 19–35 years	Continuous and categorical	≤19 20–24 25–29 30–34 ≥35	Can explain knowledge of ODS
Education level	Training courses attended by pregnant women: primary, secondary, or higher education or nothing at all	Categorical	Not in school Primary Secondary Higher	Can explain knowledge of ODS
Religion	Religious affiliation of the woman	Categorical	Muslim Catholic Protestant Kimbanguist Revival church Others, to be specified	Can explain knowledge of ODS
Marital status	Marital status of pregnant women who live in a union or alone	Categorical	Married Single Divorced Widowed	Can explain knowledge of ODS
Clinical characteristics				
Parity	Number of deliveries that the woman has experienced	Categorical	Primiparous (1 birth) Pauciparous (2–4 births) Multiparous (5–7 births) Grand multiparous (>7 births)	Can explain knowledge of ODS
Gestity	Number of pregnancies the pregnant woman had	Categorical	Too early (<19 years) Too reproductive (reproductive interval of <2 years) Too many (>5 births) Too late (>35 years)	Can explain knowledge of ODS
Antenatal consultation (ANC)	Number of times the pregnant women have attended prenatal consultation	Categorical	ANC 1 ANC 2 ANC 3 ANC 4	Can explain knowledge of ODS
Obstetric characteristics				
Antepartum (during pregnancy)	Obstetric complications that can occur in pregnant women during pregnancy	Categorical	Constant severe abdominal pain, severe vaginal bleeding, persistent fever, severe headaches or blurred vision, intense fatigue, swelling of the hands or face, difficulty in breathing, and absent or decreased fetal movement	Important variable
Peripartum (during labor and delivery)	Obstetric complications that can occur in a pregnant woman during labor or delivery	Categorical	Rupture of the membrane before the onset of labor, prolonged labor over 12 h, severe vaginal bleeding, convulsions/loss of consciousness, and retention of the placenta	Important variable
Postpartum (after childbirth)	Obstetric complications that occur in a pregnant woman after delivery	Categorical	Severe vaginal bleeding, foul-smelling vaginal discharge, severe abdominal pain, persistent fever, and intense fatigue	Important variable

**Table 2:** Operational definitions of variables.

Category	ODS	Has Knowledge of Danger Signs	Number of ODS Cited
Antepartum (during pregnancy)	Constant severe abdominal pain, severe vaginal bleeding, fever and weakness (cannot get out of bed), rapid or difficult breathing, severe headaches with blurred vision, rapid or difficult breathing, swelling of fingers, face and legs, severe fatigue, lack of or decreased fetal movement [2,4,5]	Yes	≥3 signs
		No	<3 signs
Peripartum (during labor/delivery)	Rupture of the membrane before the onset of labor, prolonged labor of >12 h, severe vaginal bleeding, convulsions/loss of consciousness, retention of the placenta... [5,7,20]	Yes	≥3 signs
		No	<3 signs
Postpartum	Severe vaginal bleeding, foul-smelling vaginal discharge, severe abdominal pain, fever, intense fatigue... [5,7,20]	Yes	≥3 signs
		No	<3 signs

**Table 3:** Sociodemographic and clinical characteristics of respondents.

Variables	N	%	Mean	Min–Max
Age (years)	623		25 (±7)	12–49
≤19 years	143	22.9		
20–29 years	303	48.9		
30–39 years	158	25.4		
40–49 years	19	3.1		
**Level of education**	**626**			
Not in school	39	6.2		
Primary	208	32.4		
Secondary	379	60.6		
Higher	5	0.8		
**Marital status**	**626**			
Single	4	0.6		
Married/common-law	622	99.4		
**Occupation**	**626**			
Unemployed	91	14.5		
Pupil/student	13	2.1		
Farmer/trader	504	80.5		
Civil servant	18	2.9		
**Religion**	**624**			
Muslim	424	67.9		
Revival church	94	15.1		
Catholic	61	9.8		
Protestant	41	6.6		
Kimbanguist	4	0.6		
**Parity**	**626**			
First pregnancy	74	11.8		
Given birth 1 time	131	20.9		
Given birth 2 times	105	16.8		
Given birth 3–5 times	130	20.8		
Given birth >5 times	186	29.7		
**Prenatal consultations ANC 1**	**626**			
Yes	355	56.7		
No	271	43.3		
**Completion of** **all 4 ANCs (completion** **of ANC** **4** **)**	**626**			
Yes	63	10.1		
No	563	89.9		

n, number of subjects; %, percentage of subjects; Mean, standard deviation; Min, minimum observed value; Max, maximum observed value.

**Table 4:** Knowledge of ODS among pregnant women.

Women Surveyed	n	% (95% CI)
Knowledge of signs		
Yes	137	21.9 (18.7–25.3)
No	489	78.1 (74.7–81.3)
Total	626	

n, number of subjects; %, percentage of subjects; 95% CI, 95% confidence interval.

**Table 5:** Knowledge of ODS according to sociodemographic and clinical characteristics of respondents.

Variables	N	% KODS	Adjusted OR (95% CI)	*p* Value
Age groups	623			0.015
≤19 years	143	14.7	1	
20–29 years	303	20.8	1.5 (0.9–2.6)	
30–39 years	158	22.1	2.4 (1.3–4.2)	
40–49 years	19	31.6	2.7 (0.9–7.8)	
**Level of education**	**626**			**0.217 ^f^**
No schooling	39	23.1	1	
Primary	203	25.6	1.2 (0.5–2.6)	
Secondary	379	19.5	0.8 (0.4–1.8)	
Higher	5	40.0	2.2 (0.3–15.4)	
**Marital status**	**626**			**0.629 ^f^**
Single/divorced/widowed	4	25.0	1	
Married/common-law	622	21.9	0.8 (0.1–36.8)	
**Occupation**	**626**			**0.122 ^f^**
Unemployed	91	13.2	1	
Pupil/student	13	14.4	1.2 (0.2–6.1)	
Farmer/trader	504	23.8	2.1 (1.0–3.9)	
Civil servant	18	16.7	1.3 (0.3–5.2)	
**Religion**	**624**			**0.099 ^f^**
Muslim	424	22.4	1	
Revival church	94	12.7	0.5 (0.3–0.9)	
Catholic	61	27.9	1.3 (0.7–2.5)	
Protestant	41	26.8	1.3 (0.6–2.6)	
Kimbanguist	4	0.0	--	
**Parity**	**626**			**0.001**
First pregnancy	74	6.8	1	
Given birth 1 time	131	16.0	2.6 (0.9–7.3)	
Given birth 2 times	105	24.8	4.5 (1.7–12.5)	
Given birth 3–5 times	130	25.9	4.7 (1.8–12.6)	
Given birth >5 times	186	28.0	5.4 (2.1–14.0)	
**Prenatal consultations ANC 1**	**626**			**0.952**
No	271	21.7	1	
Yes	355	21.9	1.0 (0.7–1.5)	
**Completion of all 4 ANCs (completion** **of ANC 4)**	**626**			**0.946**
No	563	21.9	1	
Yes	63	22.2	1.0 (0.2–1.9)	

n, number of subjects; %, percentage of subjects; KODS, knowledge of ODS; OR, odds ratio; 95% CI, 95% confidence interval; ^f^, value obtained by Fisher’s exact test.

**Table 6:** Final logistic regression model of knowledge of ODS as a function of independent variables.

Variables	Adjusted OR (95% CI)	*p* Value
**Age groups**		**0.739**
≤19 years	1	
20–29 years	0.8 (0.4–1.5)	0.438
30–39 years	1.1 (0.5–2.6)	0.862
40–49 years	1.2 (0.3–4.5)	0.755
**Parity**		**0.021**
First pregnancy	1	
Given birth 1 time	2.9 (1.0–8.1)	**0.049**
Given birth 2 times	5.4 (1.8–16.6)	**0.003**
Given birth 3–5 times	5.3 (1.7–16.9)	**0.004**
Given birth >5 times	4.9 (1.5–16.4)	**0.009**

OR, odds ratio; 95% CI, 95% confidence interval.

## Data Availability

The datasets used and analyzed during this study are available from the corresponding author upon reasonable request.

## Abbreviations

ANC	antenatal consultation
ODS	obstetric danger signs
DRC	Democratic Republic of the Congo
HZ	health zone
WHO	World Health Organization
